# The production of the first functional antibody mimetic in higher plants: the chloroplast makes the DARPin G3 for HER2 imaging in oncology

**DOI:** 10.1186/s40659-022-00400-7

**Published:** 2022-10-23

**Authors:** Maryam Ehsasatvatan, Bahram Baghban Kohnehrouz, Ashraf Gholizadeh, Hamideh Ofoghi, Dariush Shanehbandi

**Affiliations:** 1 Department of Plant Breeding & Biotechnology, Faculty of Agriculture, University of Tabriz, 51666 Tabriz, Iran; 2 Department of Animal Biology, Faculty of Natural Science, University of Tabriz, 51666 Tabriz, Iran; 3 Department of Biotechnology, Iranian Research Organization for Science and Technology (IROST), 33131 Tehran, Iran; 4 Immunology Research Center, Tabriz University of Medical Sciences, 51666 Tabriz, Iran

**Keywords:** Antibody mimetics, Chloroplast, DARPin, HER2, Molecular imaging, *Nicotiana tabacum*

## Abstract

**Background:**

Designed mimetic molecules are attractive tools in biopharmaceuticals and synthetic biology. They require mass and functional production for the assessment of upcoming challenges in the near future. The DARPin family is considered a mimetic pharmaceutical peptide group with high affinity binding to specific targets. DARPin G3 is designed to bind to the HER2 (human epidermal growth factor receptor 2) tyrosine kinase receptor. Overexpression of HER2 is common in some cancers, including breast cancer, and can be used as a prognostic and predictive tool for cancer. The chloroplasts are cost-effective alternatives, equal to, and sometimes better than, bacterial, yeast, or mammalian expression systems. This research examined the possibility of the production of the first antibody mimetic, DARPin G3, in tobacco chloroplasts for HER2 imaging in oncology.

**Results:**

The chloroplast specific DARPin G3 expression cassette was constructed and transformed into *N. tabacum* chloroplasts. PCR and Southern blot analysis confirmed integration of transgenes as well as chloroplastic and cellular homoplasmy. The Western blot analysis and ELISA confirmed the production of DARPin G3 at the commercial scale and high dose with the rate of 20.2% in leaf TSP and 33.7% in chloroplast TSP. The functional analysis by ELISA confirmed the binding of IMAC purified chloroplast-made DARPin G3 to the extracellular domain of the HER2 receptor with highly effective picomolar affinities. The carcinoma cellular studies by flow cytometry and immunofluorescence microscopy confirmed the correct functioning by the specific binding of the chloroplast-made DARPin G3 to the HER2 receptor on the surface of HER2-positive cancer cell lines.

**Conclusion:**

The efficient functional bioactive production of DARPin G3 in chloroplasts led us to introduce plant chloroplasts as the site of efficient production of the first antibody mimetic molecules. This report, as the first case of the cost-effective production of mimetic molecules, enables researchers in pharmaceuticals, synthetic biology, and bio-molecular engineering to develop tool boxes by producing new molecular substitutes for diverse purposes.

## Background

Human epidermal growth factor receptor 2 (HER2) is an epithelial growth factor receptor (EGFR)-related tyrosine kinase that is found to be overexpressed in many malignant tumors such as breast, ovarian, and gastric cancers. HER2 overexpression is associated with enhanced tumor aggressiveness and a high risk of recurrence and death [1, 2]. The high expression of the HER2 receptor on solid tumor cells and the accessibility of its extracellular domain make HER2 a suitable candidate for targeted immunotherapy as well as molecular imaging [3]. Therefore, accurate tracking and detection of HER2 expression levels in tumors is necessary to identify HER2-positive tumors that qualify the patient for targeted therapy. Currently, HER2 protein level is detected based on histological analysis of biopsied or surgically resected tissues from primary tumors by the use of immunohistochemistry (IHC), fluorescence in situ hybridization (FISH), and chromogenic in situ hybridization (CISH) [4]. However, the biopsy-based analyses are limited by the number of samples that can be taken and the heterogeneity of expression or changes in expression of HER2 during tumor progression [5–7]. Moreover, biopsies may contribute to cancer spread in some cases by disseminating cancer cells from the primary lesion to distant organs [8].

Non-invasive in vivo molecular imaging of HER2 has the potential to make repetitive measurements of the HER2 state of all the tumor sites simultaneously, the duration of disease progression and the period of treatment [9]. Two monoclonal antibodies targeting the extracellular domain of HER2, including trastuzumab and T-DM1, which have been described, can also be used as imaging agents [10, 11]. However, the traditional antibody conjugated with imaging molecules, apart from the high cost, has its disadvantages, such as low contrast, long half-life, poor tumor penetration ability, long bio distribution time, and slow clearance from plasma [12–14]. Therefore, the alternative mimetic molecule was developed to overcome these limitations while keeping the advantages of antibodies in terms of high specificity and affinity [15, 16]. Small engineered scaffold proteins such as anticalins [17], fibronectin domains [18], affibody molecules [19], knottins [20], and designed ankyrin repeat proteins (DARPins) [21] are a relatively new type of targeting probe for molecular imaging and have demonstrated high sensitivity in preclinical trials for radionuclide imaging of therapeutic molecular targets on the day of injection [22].

DARPins among small engineered scaffold proteins, has been introduced with high thermodynamic stabilities that recognize targets with specificities and affinities equal to or excel those of immunoglobulin-based proteins [21, 23]. Compared to natural antibodies, the small size of DARPins is favorable for excellent tissue/cell penetration, yielding a high tumor-to-background image contrast and resulting in rapid clearance from plasma [24]. DARPins recognizing the HER2 extracellular domain were selected from an artificial library by ribosome display [25], phage display [26], SNAP display [27], and bacterial surface display [28]. DARPin libraries were constructed with different numbers of internal repeats comprising constant framework residues as well as randomized amino acids with flanking constant N- and C-terminal capping repeats to shield the hydrophobic core of the proteins in the aqueous solution and are therefore relevant for the protein stability and solubility [29, 30]. So far, only two variants of DARPins, i.e. 9_29 and G3, have been tested as targeting probe that can be used for molecular imaging after conjugating with radionuclide and are in the clinical trial phase I [31].

DARPin G3 is the smallest of the DARPin constructs with a molecular mass of near 14–15 kDa and contains two internal repeat modules, and it does not form a tertiary structure. In isolation, it exerts no biological effects and only binds to subdomain IV of HER2 with picomolar affinity (91 pmol/L) at an epitope that does not overlap with trastuzumab [16, 32]. DARPin G3 in coupling to polyethylene glycol (PEGylation) delays the its circulation and promotes tumor uptake [16, 33]. Thus, these features make DARPin G3 an excellent candidate for HER2 imaging. DARPin G3 has previously been produced in *Escherichia coli* [16] and *Pichia pastoris* [21]*.* Despite comparatively high yields of 100–200 mg/L in *E. coli*, extracting and purifying intracellular proteins from bacteria remains a time-consuming process made more difficult by the presence of endotoxins [34]. Besides the technical challenges associated with *P. pastoris* expression, including low transformation efficiency and the laborious screening to identify high-expressing clones, practical experience with yeasts shows a high amount of product loss due to proteolytic degradation of the target protein in the medium [35]. Therefore, a move to a strong and manageable system would be advantageous.

Plants are a valuable alternative system for the large-scale production of bioactive recombinant proteins such as enzymes, vaccine components, and full-size immunoglobulins [36, 37]. Plants offer several advantages compared to traditional expression systems based on bacterial and mammalian cell culture, such as low production and capital costs, high scalability with relatively high protein yield, and increased safety for patients due to low risk of product contamination by human or animal pathogens and endotoxins [38–40]. For plant-made pharmaceutical protein production, chloroplast genetic engineering offers several advantages over nuclear transformation that make it an ideal system. These include a high level of foreign gene expression due to high copy numbers of the chloroplast genome in each plant cell, multi-gene insertion in a single transformation event, the absence of epigenetic effects, transgene containment by maternal inheritance of the chloroplast genome, and lack of gene silencing and position effects because of site-specific integration [41–43]. The chloroplasts lack the necessary machinery to carry out glycosylation, one of the most significant PTMs in eukaryotes [44]. However, some post-translational modifications such as the formation of disulfide bonds, lipidation, multimerization, and N-terminal methionine excision take place within chloroplasts, allowing the proper folding of chloroplast expressed proteins [45–48]. Therefore, chloroplast transformation can be used when glycosylation is not required for the stability or physiological activity of the expressed protein.

Previous studies have demonstrated the successful expression of a variety of therapeutic proteins in plant chloroplasts, including vaccine antigens against bacterial, viral, and protozoan pathogens [49–51], insulin-like growth factor [52], coagulation factor IX [53], human transforming growth factor-β3 [54], and SAG1 [55]. Almost all of these pharmaceutical proteins are expressed in tobacco chloroplasts. The maximum foreign protein accumulation reported for chloroplasts was up to 70% of total leaf soluble protein (TSP) in transplastomic tobacco [56, 57].

In the present work, we report on the integration and expression of the DARPin variant G3 in the tobacco (*Nicotiana tabacum* L.) chloroplasts. The correct size and accumulation level of the protein were analyzed by Western blot analysis and ELISA. We tested the chloroplast-made DARPin G3 for binding to the HER2 extracellular domain in vitro by flow cytometry. Subsequently, the immunofluorescent microscopy analysis was used to image DARPin G3 binding to cell-surface HER2. There is no reference for antibody mimetic expression in plant cells so far, and this is the first report on DARPin G3 production in plants, especially in chloroplasts. This could be a first step towards commercial production of plant-based valuable mimetic molecules.

## Results

### Vector design and construction for high chloroplastic expression

**Fig. 1:**
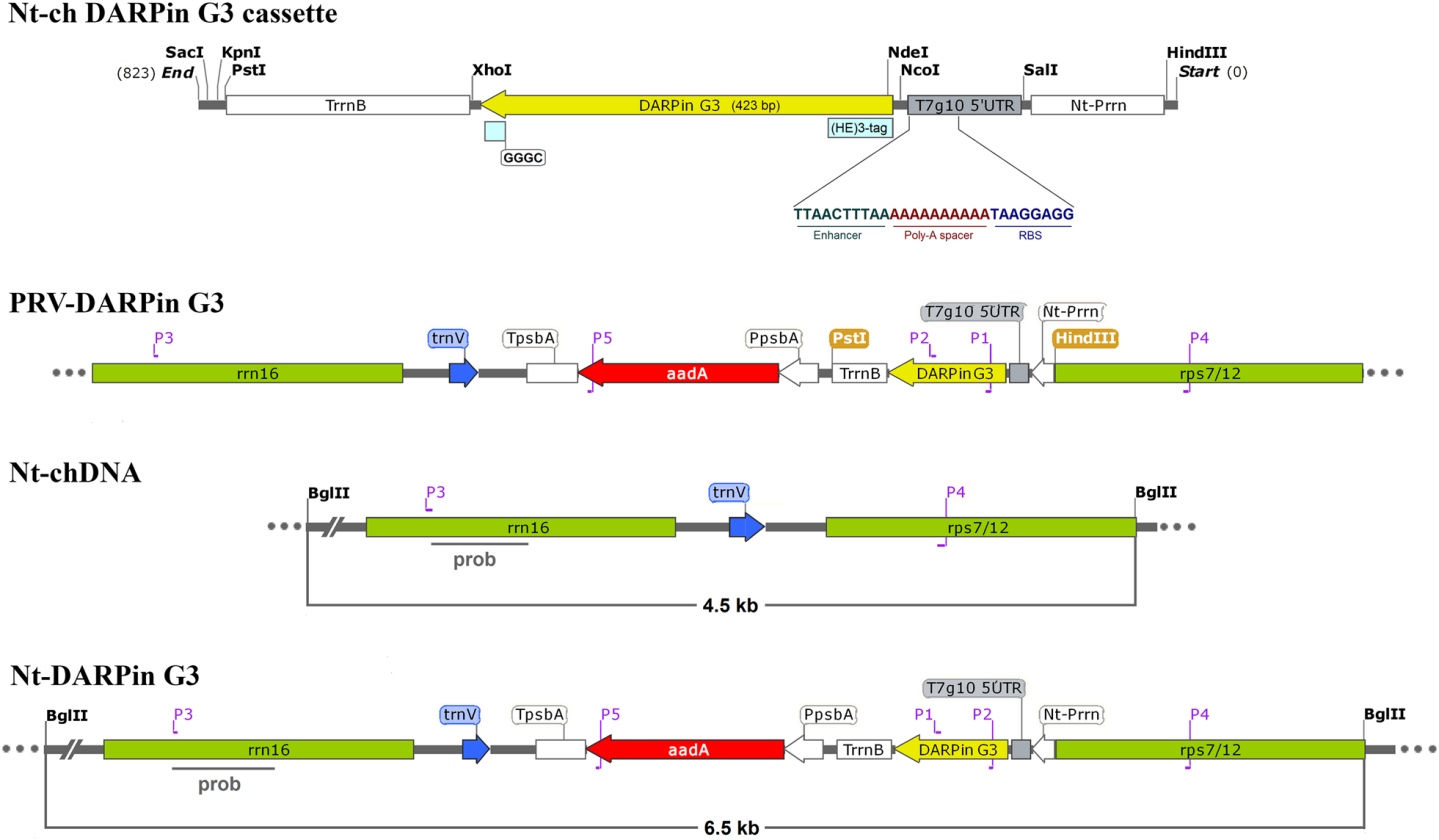
Physical map of fine structure for the chloroplast specific DARPin G3 expression cassette (NT-ch DARPin G3 cassette), plastid transformation vectors of the pPRV111A harboring DARPin G3 expression cassettes (PRV-DARPin G3), targeting region in the wild-type tobacco plastid genome (Nt-chDNA) and transformed plastid genome regions (Nt-DARPin G3). Nt-Prrn: ribosomal RNA operon promoter from tobacco; T7g10 5′ UTR: 5′ untranslated region of bacteriophage T7 gene 10; DARPin G3: coding sequence of DARPin G3, TrrnB: rrnB 3′ untranslated region from *E. coli;* PpsbA: promoter and 5′ UTR of *psbA* gene; *aadA*: aminoglycoside 3′- adenylytransferase gene; TpsbA: terminator of *psbA* gene. The transgenes are targeted to the intergenic region between the rrn16 and rps7/12 plastid genes. Primers P1/P2 land on the DARPin G3 coding sequence, generating a 215 bp fragment. Primers P3/P4 land on the rrn16 and the rps7/12 flanking sequence, generating ~ 4 kb and ~ 2 kb fragments in transplastomic and wild-type plants, respectively. The expected sizes of the DNA fragments in Southern blot analyses with the restriction enzyme *Bgl*II are indicated. The location of the Southern blotting probe is shown as a black bar

To examine and explore the potential of chloroplasts to produce small engineered scaffold proteins in higher amounts, the chloroplastic DARPin G3 expression cassette based on the pPRV111A vector [58] was designed and synthesized (Fig. 1a). The amino acid sequence for DARPin G3 was taken from a previously characterized phage display library member selected against human epidermal growth receptor 2 [59]. The encoding nucleotide sequence of DARPin G3 was deduced by codon optimization for expression in the *N. tabacum* chloroplast (Bankit Accession Number of ON186655). As shown in Fig. 1, to access the highest expression level of DARPin G3 in chloroplast, the new combination of transcriptional and translational regulatory elements were added to the optimized DARPin encoding sequence along with a histidine-glutamate (HE)_3_-tag at the N-terminal end and a cysteine-containing linker sequence (GGGC) at the C-terminal end, which is required for the further site-specific conjugation and labeling studies. The native tobacco plastid ribosomal operon (P*rrn*) promoter was used to drive the DARPin encoding sequence, the T7g10 5' UTR was used to boost translation, and the transcript was stabilized by the *E. coli rrnB* 3′ UTR. The epsilon sequence (5-UUAACUUUAA) as a translation enhancer from gene 10 of the T7 phage was placed upstream of a robust Shine-Dalgarno sequences following a poly-A sequence as a spacer that promotes the expression of downstream genes of any ORF [60].

The pPRV111A vector contains the *aadA* gene (aminoglycoside 3′-adenylyltransferase), which confer resistance to both spectinomycin and streptomycin. The *aadA* gene is driven by the *psbA* promoter to inhibit developing untransformed chloroplasts in favor of antibiotic-resistant plantlet regeneration and increasing homoplasmy. The *rrn16* and *rps7/12* flanking sequences were included in the pPRV111A vector for integration via double homologous recombination in the inverted repeats of IRA and IRB in the chloroplast genome, resulting in double gene dosage in comparison to small and large single-copy regions. The constructed expression vector called PRV::DARPin G3 was used to generate DARPin G3-expressing transplastomic tobacco plants. The complete description of transferred genes within the tobacco chloroplast genome, together with the used primers to validate transgene insertion and the used probe for Southern blot analysis, are shown in Fig. 1.

### Analysis of the structural model

**Fig. 2:**
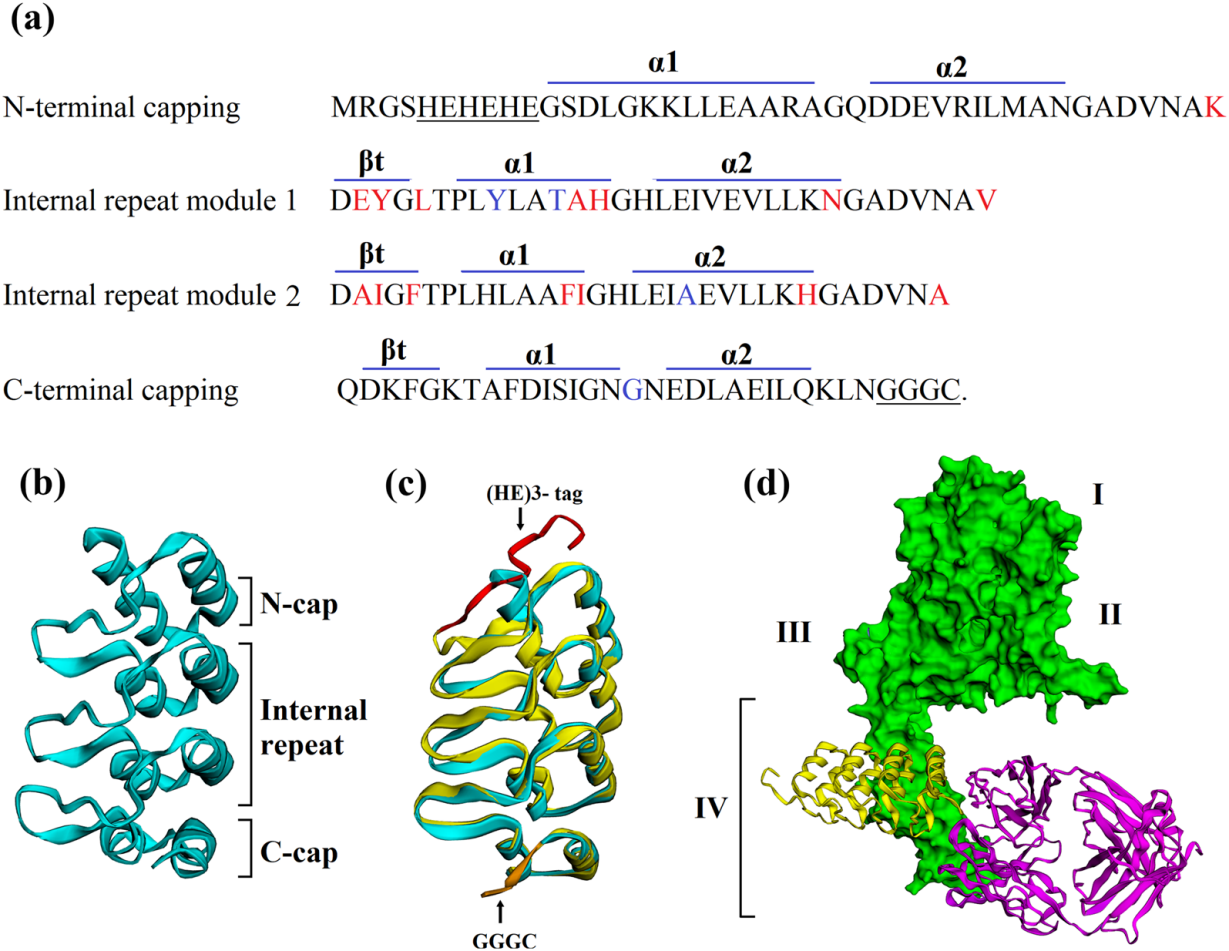
Sequence, structure, and structural model of chloroplast-made DARPin G3 in complex with the HER2. **a** The chloroplast-made DARPin G3 N-terminal capping ankyrin repeat, two designed ankyrin repeat module, and C-terminal capping ankyrin repeat amino acid sequences. The secondary structure components (α-helices and β turns) are shown above their respective sequences. The designed ankyrin repeat modules consist of 26 defined framework residues and 7 randomized residues (6 potential interaction residues and 1 framework residue) shown in red. Also, the DARPin G3 has four mutations at defined framework positions, which are shown in blue. A (HE)_3_-tag for purification at the N-terminus and a cysteine containing linker at the C-terminus were underlined. **b** The 3D structure of DARPin G3 (PDB id: 2JAB), and **c** The modeling structure of chloroplast-made ((HE)3-tag was colored red, and cysteine-containing linker was colored orange). The backbones of the DARPin G3 are colored in cyan, which are superposed with the homology model of chloroplast-made DARPin G3 in yellow. **d** Structural model of the chloroplast-made DARPin G3 (yellow) in complex with the human epidermal growth factor receptor 2 HER2 (green). In magenta, the structure of trastuzumab Fab bound to HER2 is shown superimposed on the G3-HER2 complex

The chloroplast-made DARPin G3 structurally is comprised of three parts. Its N-terminal and C-terminal differ from original DARPin G3. The N-terminal capping repeat equipped with a (HE)_3_-tag, two internal designed ankyrin repeat modules, and C-terminal capping repeat linked with a cysteine-containing sequence. Each internal repeat consists of 33 amino acids, forming a β-turn followed by two anti-parallel α-helices and a loop that binds to the β-turn of the next ankyrin repeat. In each of the ankyrin repeats, 26 amino acids have defined framework residues, six amino acids considered as random target interaction residues, and one randomized framework residue (usually either histidine, tyrosine, or asparagine). There is no cysteine residue in this molecular tool, which enables engineering to fuse any particular peptide or chemicals to the molecule. The affinity of DARPin G3 for HER2 increased by four further mutations at framework positions in comparison to the consensus variants (Fig. 2a–c).

Trastuzumab and the DARPin G3 bind to contiguous but non-overlapping epitopes of subdomain IV in the HER2 extracellular domain, indicating that they do not compete for HER2 binding. The structural modeling of the protein–protein interactions of chloroplast-made DARPin G3 and HER2 extracellular domain confirmed this non-overlapping binding (Fig. 2d). Moreover, it revealed that the chloroplast-made DARPin G3 has no interaction with HER2 domains I-III. All structural modeling results on the chloroplast-made DARPin G3 are in agreement with previous works done in *E. coli* and yeast production systems [29, 61, 62].

### Generation and evaluation of DARPin G3 producing transplastomic tobacco

**Fig. 3:**
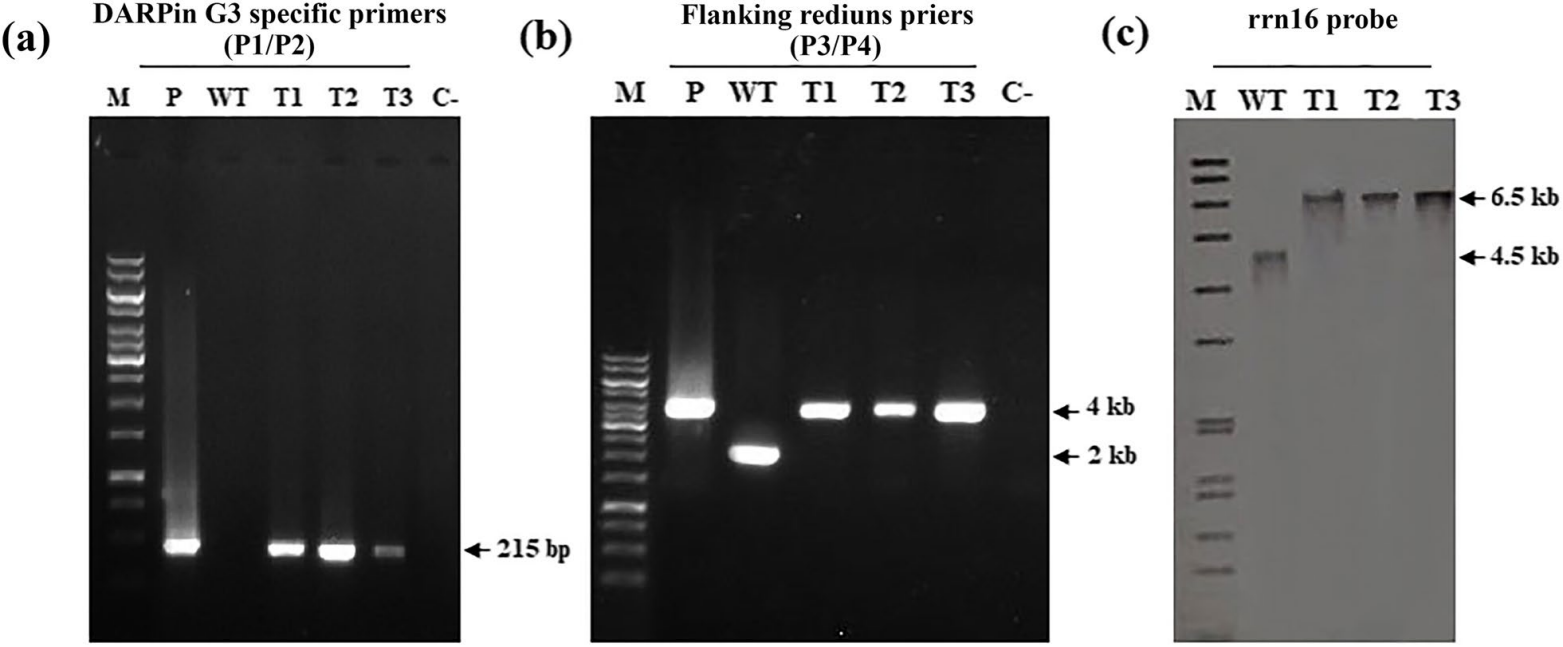
Molecular analysis of transplastomic plants. The transplastomic nature of the plants was confirmed by PCR analysis with **a** DARPin G3-specific primers (P1/P2) and **b** flanking sequence primers (P3/P4). M: 1 kb DNA sizer marker P: PRV-DARPin G3 plasmid as positive control; WT: wild-type plant; T1-3: three independent transplastomic plants; C¯: DNA free reaction as the negative control. **c** Southern blot confirms DARPin G3 gene integration and homoplasmy in three transplastomic plants. Transplastomic plants had one 6.5 kb fragment as expected for homoplasmic transformed plants, whereas the wild-type plant had a 4.5 kb fragment after DNA was digested with *Bgl*II. M: Dig-labeled molecular marker VII; WT: wild-type plant; T1-3: three independent transplastomic plants

The DARPin G3-expressing transplastomic tobacco plants were generated by the bombardment of leave tissues using a particle delivery system (PDS-1000/He). To reach the homoplasmy in transplastomic flowering tobacco plants, plantlet regeneration was carried out thrice under stringent spectinomycin/streptomycin selection pressure, and the resulted putative homoplasmic plants were analyzed by both PCR and Southern blot hybridization. The PCR results of total cellular DNA from three putative transplastomic plants showed a 215 bp fragment amplification corresponding to a part of the DARPin G3 gene using DARPin G3 gene-specific primer pairs (P1/P2) (Fig. 3a). The integrity of the transferred transgenes and homoplasmy of DARPin G3 expressing three transplastomic plants was confirmed using primer pairs from *rrn16* and *rps7/12* flanking sequences (P3/P4). A 4 kb PCR-amplified product were detected in all transplastomic plants, while only a 2 kb PCR-amplified product was detected alone in the wild-type plant (Fig. 3b). This indicated that all three transplastomic plants were 100% homoplasmic for the transgene insertion. The *Bgl*II digested cellular total DNA from three PCR positive transplastomic lines was subjected to Southern blot hybridization using a *rrn16* probe to further verify the correct integration of the DARPin G3 gene and homoplasmic status (Fig. 3c). The homoplasmy of three transplastomic plants was confirmed by the presence of a single signal as the ~ 6.5 kb fragment in transplastomic plants, compared to the ~ 4.5 kb fragment from the wild-type plant. The absence of ~ 4.5 kb hybridizing fragment in all transplastomic plants confirmed that 100% homoplasmy for DARPin G3 have been successfully achieved, typically after three regeneration cycles.

### Verification of successful expression and quantification of chloroplast-made DARPin G3

**Fig. 4:**
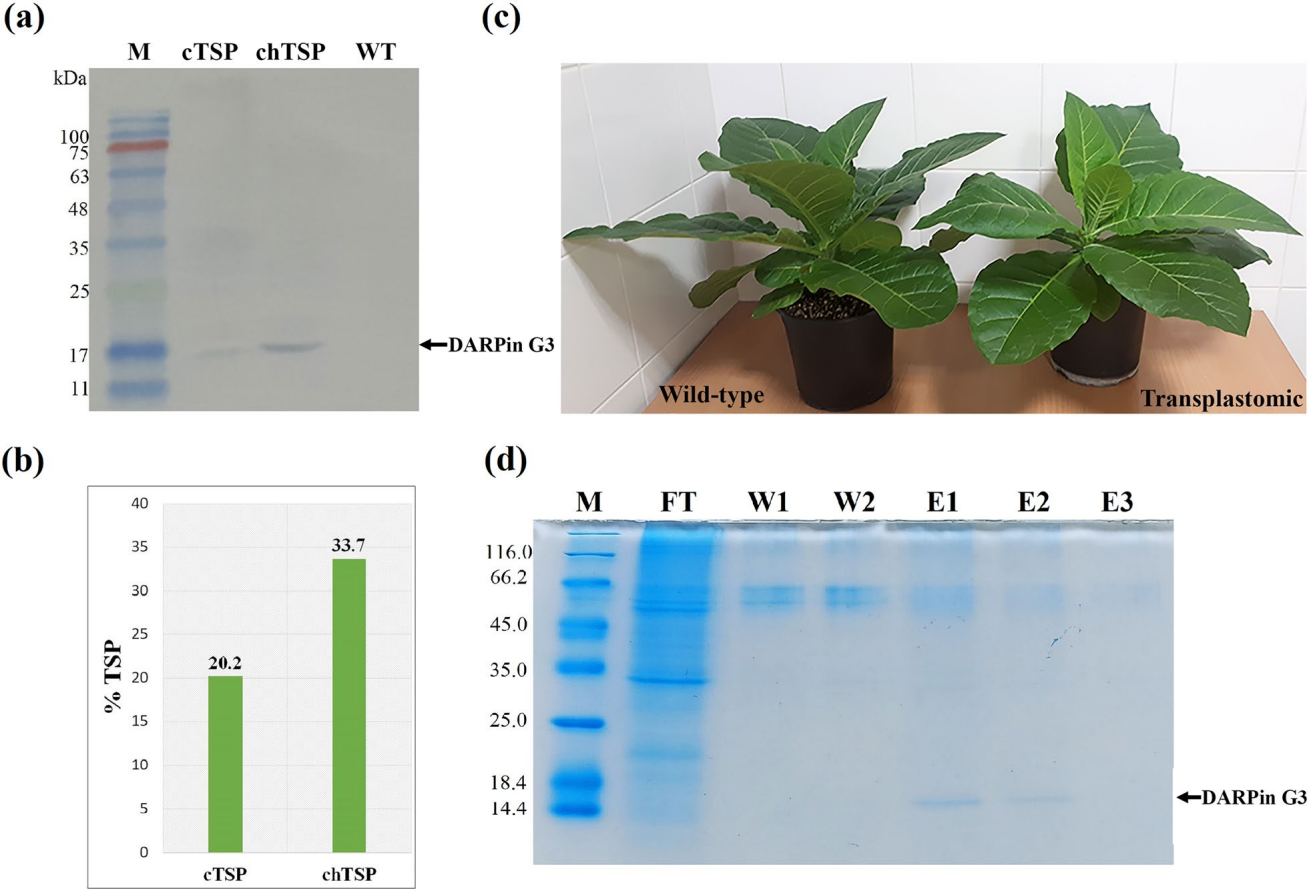
Accumulation of DARPin G3 in *N. tabacum* chloroplasts and purification by the (HE)_3_-tag. **a** Western blot analysis of chloroplast-made DARPin G3 expression in tobacco transplastomic plants. Blots were detected using rabbit anti-His-tag antibody as a primary antibody and goat anti-rabbit conjugated with HRP antibody as a secondary antibody. M: prestained molecular mass markers (kDa); cTSP: Total soluble protein from transplastomic plant leaves; chTSP: total soluble protein from isolated chloroplasts of the transplastomic plants; WT: total soluble protein from wild-type plant. Sizes are shown on the left-hand side in kilodaltons (kDa). **b** ELISA analysis for the DARPin G3 content expressed in tobacco chloroplast. Wild-type tobacco protein extracted by the same procedure was used as a negative control. A purified 15 kDa His-tagged protein was used as a positive standard. Rabbit anti-His-tag antibody (1:2000) and goat anti-rabbit antibody (1:10,000) were used as primary and secondary antibodies, respectively. Detection was performed with the TMB substrate for 30 min at RT. The measurement was performed in biological triplicates. **c** Apparent morphological equivalency between wild-type (left) and transplastomic (right) tobacco plants. **d** SDS–PAGE analysis of purified chloroplast-made DARPin G3 from the total soluble protein of isolated intact chloroplast from transplastomic plants. 12% Tris–Glycine gel stained with Coomassie blue; samples were processed with 2 × SDS–PAGE reducing buffer. M: unstained protein MW marker (Thermo scientific); FT: flow-through from Ni^+^ NTA resin; W1, W2: wash steps; E1–E3: three eluents from IMAC. Purified chloroplast-made DARPin G3 is marked on the gel by an arrow

The Western blot analysis was carried out on both cellular total soluble protein (cTSP) and chloroplastic total soluble protein (chTSP) for analysis of protein accumulation and integrity in transplastomic plants. As shown in Fig. 4a, the selected transplastomic plant expressed a protein with a molecular weight of ~ 15 kDa, corresponding to the calculated size of the chloroplast-made DARPin G3 protein. In spite of this, no such bands were detected in Western blot analysis of wild-type tobacco plants as a control (Fig. 4a).

The enzyme-linked immunosorbent assay (ELISA) was used to more accurately quantify the DARPin G3 protein expressed in tobacco chloroplasts. The amount of chloroplast-made DARPin G3 protein in the transplastomic plants was calculated as its percentage in cTSP and chTSP by triplicate ELISA. The DARPin G3 protein content was estimated 20.2% of cTSP and 33.7% of chTSP in the transplastomic plants (Fig. 4b). Since the chloroplast proteins comprise the major component of the plant cell proteins, representing 40% of total cellular protein [63], the ELISA results on the accumulation of DARPin G3 protein in chloroplasts and cytoplasm reconfirmed this ratio again. As expected, the wild-type plant did not develop any color signal in the ELISA test, indicating the complete absence of DARPin G3 protein.

The morphological equivalency of wild-type tobacco with transplastomic tobacco showed that the expression and accumulation of very high levels of chloroplast-made DARPin G3 in transplastomic tobacco had no apparent negative effect on plant growth or development (Fig. 4c), indicating physiologically no interfere occurred between chloroplast-made DARPin G3 in the plastidial metabolism or gene expression.

### Purification of chloroplast-made DARPin G3

To provide the required amounts of chloroplast-made DARPin G3 for further analysis on HER2 binding, it was purified from the total soluble protein of isolated chloroplasts using Ni^+^-NTA agarose resin. The chloroplast-made DARPin G3 protein was produced along with a negatively charged and hydrophilic N-terminal histidine-glutamate (HE)_3_-tag. The principle of IMAC purification is based on that the histidine-tag containing proteins have an affinity for certain metal ions like Co_2_^+^ and Ni_2_^+^ that can be immobilized on a chromatographic matrix, ensuring specific capture. Although the low concentration imidazole washings are commonly used before protein elution to limit non-specific protein binding, it increased (HE)_3_-DARPins losses from the IMAC columns [21]. Therefore, we used the binding and washing buffer without imidazole. The Coomassie stain of the purified proteins separated by SDS–PAGE showed that chloroplast-made DARPin G3 had been separated from other chloroplastic soluble proteins. The SDS–PAGE assay of purified protein shows that the first elution fraction contains the highest amount of chloroplast-made DARPin G3 protein. In the third fraction, there is probably a very small amount of target protein that is not visible in the Coomassie blue stained gel, and only the more sensitive silver nitrate-staining method made it visible (Fig. 4d). Meanwhile, the 55-kDa large subunit of Rubisco was slightly present in all fractions. This is common because Rubisco is the most abundant protein in plants and possibly the most abundant protein on the planet [64]. Removing it from the IMAC purified protein necessitates additional purification steps. As indicated in Fig. 4d, a minimal number of proteins were observed in the wash fractions, too.

### HER2 binding capacity of chloroplast-made DARPin G3

**Fig. 5:**
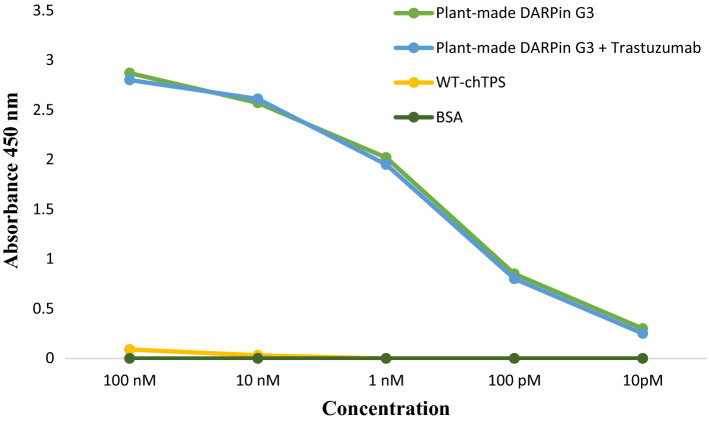
Binding affinity of chloroplast-made DARPin G3 to the extracellular domain of HER2 measured by ELISA. Polystyrene 96 wells plate were coated with 100 μg of extracellular domain of HER2 and incubated with serially diluted chloroplast-made DARPin G3. An HRP-conjugated anti-His tag antibody was used for detection. Experiments were performed in triplicate and crude extract of isolated chloroplasts of wild-type plant and BSA served as a negative control

An ELISA was performed using HER2 coated plates to determine whether the chloroplast-made DARPin G3 as a potent antibody mimetic would be capable of binding to HER2 as the target antigen or not. The binding affinity test by ELISA using the purified chloroplast-made DARPin G3 exhibits this capacity for HER2 in the sub-nanomolar range (Fig. 5). This range for the chloroplast-made DARPin G3 is sufficient affinities compared to previously reported studies on *E. coli* [59] and *P. pastoris* [21]. In addition, the ELISA results showed the chloroplast-made DARPin G3 did not compete for the same epitope with trastuzumab (Fig. 5). The total soluble protein isolated from the wild-type chloroplasts as well as BSA were used as a negative control, indicating no binding to HER2, at all.

### Chloroplast-made DARPin G3 specificity for HER2

**Fig. 6:**
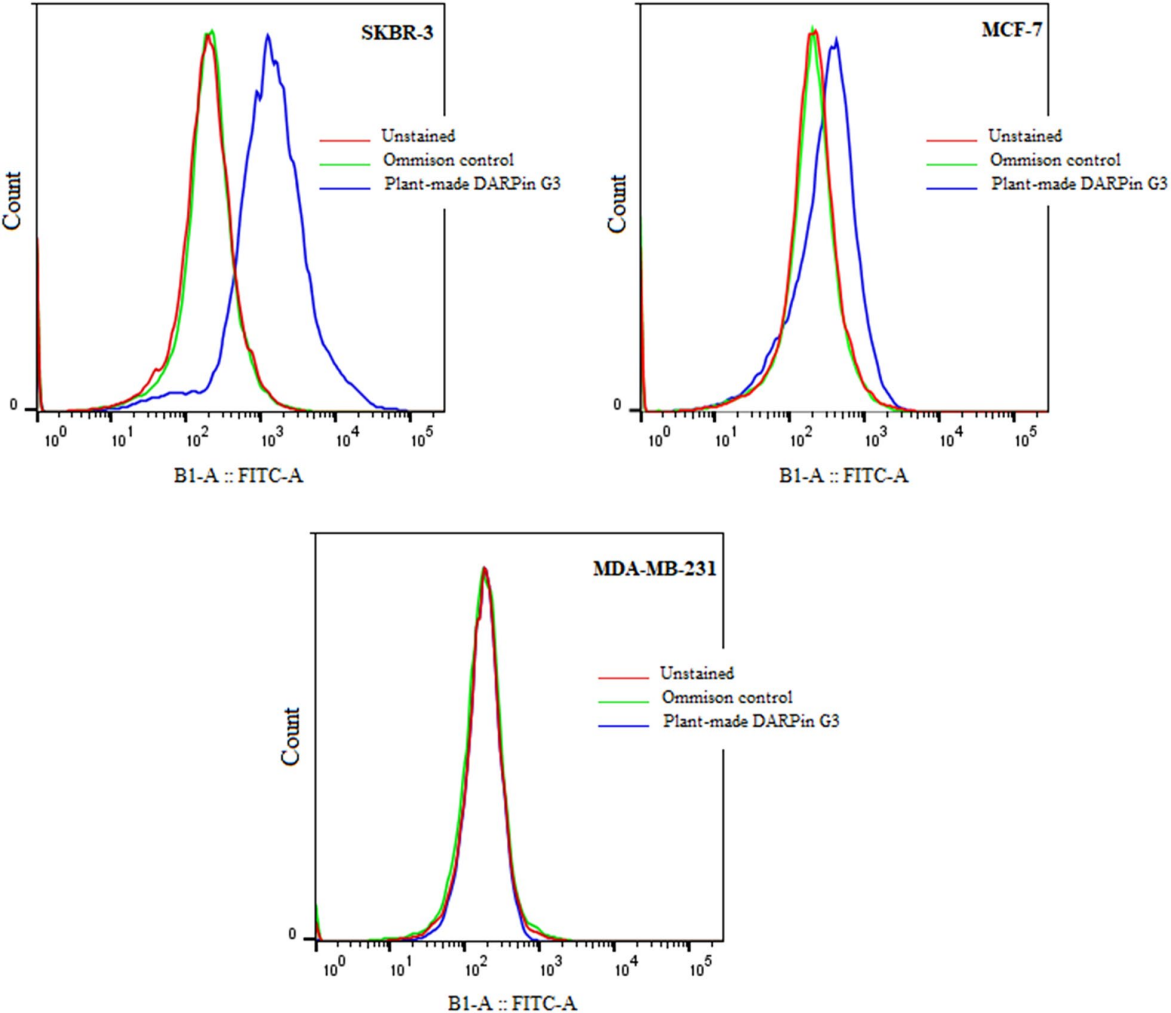
Binding of chloroplast-made (HE)_3_-DARPin G3 to HER2 on different cell lines by analytical flow cytometry. Binding was detected via His-tag specific and fluorescein isothiocyanate-coupled (FITC) antibodies. The fluorescence intensity logarithm of counted cells was plotted versus the cell count. Since all cell samples were homogeneous cell populations, the logarithm of the average fluorescence intensity was plotted as a single value for better comparison. Omission control cells were treated with antibodies in the absence of DARPin G3. Cells were incubated with PBS or anti-His-tag antibody followed by FITC conjugated antibody to serve as negative controls. Data from representative experiments are shown

To check whether the chloroplast-made DARPin G3 could also bind to the HER2 on the cell surface, we employed three human breast cancer cell lines known to express HER2 at different levels [65]. The SKBR-3 line has shown to overexpress HER2 strongly, MCF-7 shown as weakly overexpress HER2, and MDA-MB-231, which has not seen to overexpress HER2. To have the maximum stringency in the binding reaction, the chloroplast-made DARPin G3 was allowed to bind to the cells in solution at a relatively low concentration of 100 nM. A primary rabbit anti-His-tag antibody and a secondary fluorescein isothiocyanate-coupled (FITC) anti-rabbit antibody were used to detect binding in a fluorescence-activated cell sorter. Flow cytometry analysis of the three treated breast adenocarcinoma cell lines revealed that chloroplast-made DARPin G3 bound to HER2-positive SKBR-3 cell line but not to MDA-MB-231 as a HER2-negative human breast adenocarcinoma cell line. Binding to MCF-7 cells was highly reduced compared to SKBR-3 (Fig. 6). These results are in agreement with the formerly described HER2 expression patterns of these cell lines [65].

**Fig. 7:**
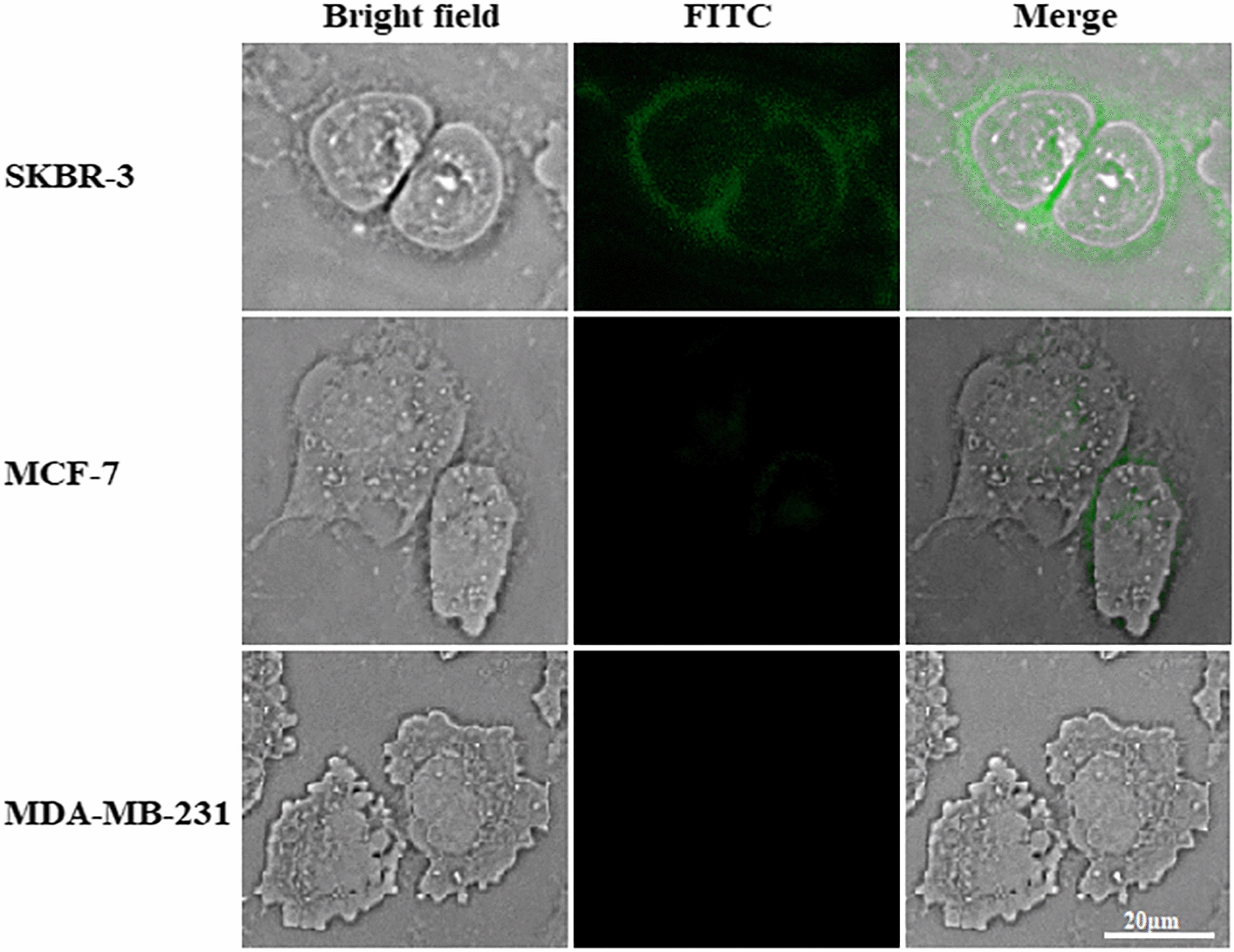
Binding of chloroplast-made (HE)_3_-DARPin G3 to HER2 on different cell lines by immunofluorescent microscopy. Cells were incubated with chloroplast-made DARPin G3 followed by a FITC conjugated antibody which showed the HER2 specific targeting on the cell surface. Laser power and gain were kept constant, and brightness and contrast settings were adjusted equally. The scale bar represents 20 μm

Fluorescent microscopy was also used to visualize cellular binding of the chloroplast-made DARPin G3 to HER2 on the cell surface using FITC tags. We observed a significantly increased signal of FITC staining throughout the cell membrane in SKBR-3 cells, whereas such a signal was nearly absent in MDA-MB-231 cells (Fig. 7). The only slight signal was observed in the MCF-7 cell line. Here, the control experiment without the DARPin G3 or FITC conjugated antibody showed no FITC staining at all. Some intracellular signals indicate that at least a fraction of chloroplast-made DARPin G3 proteins has been internalized as a result of HER2 internalization, which has been previously described [24, 66, 67]. Thus, flow cytometry and immunofluorescent microscopy analysis confirmed the HER2 specificity of chloroplast-made DARPin G3 in vitro.

## Discussion

Designed mimetic scaffold/binding molecules are of special importance due to their many applications in various research and industries, including the field of synthetic biology as new research tool and clinical applications including oncology, immuno-oncology, ophthalmology, and immunology and their required mass production could be one of the upcoming challenges in the near future.

By this report for the first time, the successful expression of a scaffold protein/antibody mimetic is described as the plant-made DARPin G3 for HER2 bioimaging agent in the chloroplast system. The human epidermal growth factor receptor family, especially HER2 drives some oncogenic processes, including proliferation and invasion, and plays a significant role in the development and progress of a number of cancers [68]. Overexpression of HER2 is associated with offensive tumor behavior and poor clinical consequences and can be linked as a prognostic factor in the prediction of treatment with the anti-HER2 monoclonal antibodies of trastuzumab and pertuzumab as well as a target for molecular therapies [69, 70]. Currently, tumor biopsies are used to determine the HER2 status, but this may not accurately reflect the larger tumor mass or additional metastatic sites, increasing the risk of misclassification and the use of less effective medication. Although detecting cancer biomarkers in tissue samples can help with prognosis and treatment planning, the ability to non-invasive in vivo tumor imaging is emerging as an important tool in cancer diagnosis [71, 72].

DARPins are promising as a radionuclide molecular imaging probes because of their high affinity, small size, and lack of any toxic Fc region. This combination allows for much greater tumor penetration while also allowing for quick clearance from targeted tumor and surrounding tissues, improving both the sensitivity and specificity of visualization [72]. In order to in vivo imaging of HER2, DARPin 9–29 and DARPin G3, have been developed [21, 73]. A side-by-side comparison of tumor targeting and biodistribution of DARPins 9–29 and G3 demonstrated that DARPin G3 provides higher tumor uptake and tumor-to-organ ratios due to its smaller size and higher affinity in comparison to 9–29, which results in a preferable candidate for HER2 imaging [74].

DARPin G3 consists of four ankyrin repeats (N- and C-capping repeats, and two internal repeats) and binds to the subdomain IV of the HER2 extracellular domain. DARPin G3 and trastuzumab bind to HER2 independently, indicating that they bind to non-overlapping epitopes of subdomain IV in the HER2 extracellular domain, and that they do not compete for HER2 binding [21]. Thus, all new patients and those receiving concurrent trastuzumab may be imaged using DARPin G3 without the need for a treatment interruption [21, 75]. Pertuzumab binds to subdomain II in the extracellular domain of HER2, allowing it to bind in the presence of DARPin G3. Results of macromolecular docking on chloroplast-made DARPin G3 with HER2 complex with trastuzumab fab are in agreement with structural modeling and experimental observations that trastuzumab and DARPin G3 binding to HER2 are not competitive with each other [61, 75, 76].

Since the commercial production of recombinant proteins is limited by their high production costs and the difficulty of extracting and purifying them, plant-based expression systems could be attractive because they are often less expensive and can yield high levels of protein expression. Moreover, in the plant-based expression systems, folding and modification of proteins are more similar to human processes [77, 78]. In recent years, a variety of recombinant proteins have been successfully expressed in plants, especially in chloroplasts, with similar biological activities compared to those produced by traditional expression systems [42, 49, 52, 79–81].

According to this exploration study, we successfully produced an ankyrin repeat protein (DARPin) variety G3 against the extracellular domain of HER2 in *N. tabacum* chloroplasts for the first time. The tobacco plant was selected to be used for the expression of DARPin G3 in this research, because of its high potential for producing more than 90 tons of leaf biomass per hectare using 20,000 plants across three harvests. Furthermore, a tobacco plant can produce up to one million seeds, which would be enough to plant over 250 hectares, allowing for quick large-scale production [82]. Moreover, it is notable that one fully grown tobacco plant is thought to be capable of producing more recombinant protein than a 300-L *E. coli* fermenter [83].

In summary, PCR products generated from unique primer sets annealing to the native chloroplast genome and or the transgene cassette confirmed correct site-specific integration of the DARPin G3 gene into the chloroplast genome. The required 100% homoplasmy of the transplastomic plants for stable cytoplasmic inheritance was also determined by the absence of a wild-type 4.5 kb hybridizing fragment in Southern blots. The higher amounts of chloroplast-made DARPin G3 accumulation by 20.2% of total soluble protein in transplastomic tobacco leaves and 33.7% of total soluble protein in chloroplasts answers all economic concerns behind the coming research consuming age of mimetic molecules and their cost-effective production by this method. This road could be paved and ensure the mass production of any new kinds of mimetic molecules needed in synthetic biology-based industries.

The results from the current study show that the chloroplast-made DARPin G3 binds HER2 with an excellent affinity similar to what has previously been reported for this DARPin expressed in *E. coli* [16] or *P. pastoris *[21] as shown using an enzyme-linked immunosorbent assay (ELISA). Flow cytometry and immunofluorescent microscopy also revealed the strong document on HER2 specific binding of chloroplast-made DARPin G3 on the adenocarcinoma cell surface too.

Many factors can influence the level of foreign protein accumulation in transgenic plastids, including protein type, transcription and translation regulatory elements, plant tissue, plant growth stage, insertion site in the chloroplast genome, and RNA and protein stability [84]. Among these parameters, new combination of transcriptional and translational elements were designed and used in chloroplast expression construct. So, the high-level expression of DARPin G3 in tobacco chloroplast was achieved by the designed construct consisting of T7 gene 10 leader contains a strong Shine-Dalgarno sequence, an epsilon motif as an enhancer sequence, and a 10-nucleotide poly-A-spacer between the epsilon motif and the ribosomal binding site. This chimeric 5′ UTR was combined with the strong ribosomal RNA operon promoter (P*rrn*) and *E. coli rrnB* 3′ UTR.

At the transcriptional level, the most essential aspect of enhancing transgene expression is the use of a proper promoter. The promoter driving the ribosomal RNA operon, P*rrn*, is the strongest promoter in higher plant plastids and works without any transcriptional factors or activators. P*rrn* was used in the majority of studies reporting high levels of plastid transgene expression [56, 85–87].

At the translational level of expression, one of the key factors affecting the level of expression of a particular plastid transgene is the choice of the 5′ UTR [88, 89]. A wide range of 5′ UTRs have been examined in transplastomic experiments, largely to assess how well they promote reporter gene translation [90, 91]. The T7g10 5ʹ UTR sequence has been used in many studies to increase the accumulation of heterologous proteins in plastids [86, 92–95]. This sequence results in the highest level of heterologous protein accumulation in the plastid when placed after a strong promoter sequence of ribosomal RNA operons (P*rrn*) [56, 85].

For nearly three decades, the involvement of plastidial 3′ UTRs in mRNA stability has been mentioned, and the choice of the 3′ UTR could influence RNA accumulation levels. Researchers have determined that bacterial and plastid 3′ UTRs seem to be similar [96, 97], and Tangphatsornruang et al. (2011) reported that in tobacco transplastomic plants, the *E. coli rrnB* 3′ region resulted in higher levels of *gfp* transcript accumulation than the tobacco plastid *psbA*, *rpoA*, *rbcL*, and *petD* 3′ UTRs [98]. We used the bacterial 3′ UTR as a bipartite terminator in our cassette, showing the superiority of the T*rrnB,* which may contribute to the high chloroplast-made DARPin G3 expression level in our lines.

In spite of previous works on DARPin G3, the histidine-glutamate (HE)_3_-tag inserted on the N-terminal of the chloroplast-made DARPin G3 due to the results of studies assessing the composition and position influence of histidine-containing tags on the biodistribution of DARPin G3 in normal tissue, demonstrating that the hydrophilic tags at the N-terminus provides proper biodistribution of ^99m^Tc labeled DARPin G3, reduce background liver uptake and result in the best imaging properties [21, 73] while still permitting tag-mediated IMAC purification [99, 100]. The usage of the (HE)_3_-tag in Affibody [101–103], as well as short peptides [104] reduced the hepatic uptake. Significantly, NCBI BLAST search for a HEHEHE as the query, could found almost identical sequences such as HEHEH in the zinc transporter, EHEHE in tubulin polyglutamylase, and HEHEQE in kinase 3 which appeared to be present in the human proteins. The occurrence of such similar sequences in human proteins indicates that the (HE)_3_-tag could be a potentially safer alternative to the His_6_-tag. Because of the weaker interaction between Co_2_^+^ and the (HE)_3_-tag, Ni_2_^+^-containing resin was used in this study to increase the (HE)_3_-tag binding capacity.

Moreover, a unique cysteine was introduced through a triglycine spacer GGGC at the C-terminus of the protein. As the DARPin scaffolds are cysteine-free, the thiol group of the engineered cysteine can be used for site-specific thiol-directed cytotoxic drug conjugation, radio-labeling via a bifunctional chelator, as well as PEGylation of the DARPins [16, 24]. Importantly, insertion of the C-terminal cysteine did not alter the chloroplast-made DARPin G3 affinity for HER2. Earlier studies on Affibody molecules have demonstrated that cysteine-containing variants retain high affinity for HER2, regardless of the composition and position of the histidine-based tags [99].

## Conclusions

This study demonstrates the producibility of functional DARPin G3 as the first antibody mimetic and scaffold protein in the chloroplast expression system. Although the potential of chloroplasts in the production of biopharmaceuticals has already been proven, the artificial molecular substituents with diverse applications are also produced as containment biomaterial inside plant cells. Apart from therapeutic or diagnostic applications, these mimetic molecules are required as research tools as ex-vivo or in-vivo designed biological circuits.

Access to mass production of any mimetic molecules in plants using a plastidial-based platform opens up a new area in the coming age of synthetic biology for fast and accurate assessment and examination of any mimetic biopharmaceuticals or biologically active substituents. All mimetic antibodies and more different synthetic binding and scaffold proteins as members of any combinatorial library could be produced under this platform. This provides scientists with the means to meet their own requirements for biologically or industrially fine, accurate, and active alternatives as well as structurally substituting molecules.

## Materials and methods

### Construction of chloroplast expression vector

The chloroplast transformation vector pPRV111A (Fig. 1, GenBank Accession number U12812.1) [58] was used to clone the DARPin G3 gene in the study. The pPRV111A vector contains the *aadA* coding sequence and is expressed under the *psbA* chloroplast gene's promoter (P*psbA*) and terminator (T*psbA*) regulatory regions, which confers resistance to both spectinomycin and streptomycin. Multiple cloning sites are flanked by *rrn16* and *rps7*/*12* tobacco plastid DNA homologous sequences in this vector to target the insertion of the transgenes into the plastid genome's inverted repeat regions via double homologous recombination.

The DARPin G3 gene nucleotide sequence was deduced from a previously characterized DARPin G3 amino acid sequence deposited in PDB (PDB id: 2JAB), taking into account the codon usage in high expression in *Nicotiana tabacum* chloroplast was achieved with backtranseq online software (https://www.ebi.ac.uk/Tools/st/emboss_backtranseq/). The additional histidine-containing sequences ((HE)_3_-tags) and a cysteine-containing sequence were added to the *N*- and *C*-terminus of the DARPin G3 gene, respectively. The amino acid sequence for (HE)_3_-DARPin G3-GGGC was as follows: MRGSHEHEHEGSDLGKKLLEAARAGQDDEVRILMANGADVNAKDEYGLTPLYLATAHGHLEIVEVLLKNGADVNAVDAIGFTPLHLAAFIGHLEIAEVLLKHGADVNAQDKFGKTAFDISIGNGNEDLAEILQKLNGGGC.

The DARPin G3 expression cassette was constructed by fusing the coding region with the strong plastidial promoter of the rRNA operon (P*rrn*) and a T7g10 5′ UTR-derived leader sequence [105] at the 5′-end and the *rrnB* 3′-UTR from *E. coli* [98] at the 3′-end. 5′ UTR sequence contains a strong ribosome binding site (UAAGGAGGUG) [106], the epsilon motif UUAACUUUAA as an enhancer sequence [60, 107], and a 10 nucleotide poly-A-spacer between the epsilon motif and the RBS [106], which contributes to increasing the efficiency of translation initiation. The 823 bp cassette encoding DARPin variant G3 with translation control elements was chemically synthesized (Genscript, USA), digested with *Hind*III and *Pst*I, and ligated into pPRV111A cut with the same enzyme (Fig. 1). The chloroplast specific-plasmid vector harboring *aadA* and DARPin G3 encoding genes was purified and used for the transformation of *Nicotiana tabacum* chloroplasts by the biolistic method.

### Structural modeling of chloroplast-made DARPin G3

To track the structural changes of chloroplast-made DARPin G3 equipped with a (HE)_3_-tag at the N-terminal and a cysteine linker at the C-terminal, homology modeling was performed using I-TASSER [108, 109] for protein secondary structure prediction and template-based tertiary structure. Then the best scoring model was considered further. The RCSB PDB database (PDB id: 2JAB) was utilized to retrieve the DARPin G3 structure, which was then used as a target for homology modeling. PyMOL (The PyMOL Molecular Graphics System; Version 2.3.2_81) was used to align the structures of two proteins. For the protein–protein docking, the HER2 structure (i.e., ErbB2) in complex with trastuzumab fab (PDB id: 1N8Z) and the predicted model of chloroplast-made DARPin G3 were used as starting structures. For the rigid body docking of these two protein structures, we used the macromolecular docking program ZDOCK 3.0.2 [110]. In our docking, we designated the HER2 complex with trastuzumab fab as the receptor and the chloroplast-made DARPin G3 as the ligand. All structures were visualized in EzMol [111].

### Plant growth, biolistic transformation, and regeneration

To obtain the sterile in vitro tobacco plants for shooting, surface-sterilized *Nicotiana tabacum* cv. Perega seeds were germinated under sterile conditions on MS salts [112] and B5 vitamins containing 3.0% sucrose and 0.7% plant agar at 25 °C under a photoperiod of 16-h light/8-h dark. For chloroplast transformation, the mature dark green leaves excised from the in vitro tobacco plant were placed abaxial side up, on the RMOP shoot regeneration medium (MS salt, BAP, 1.0 mg/l, NAA 0.1 mg/l, 0.1 g myoinositol, 30 g sucrose, pH 5.8, and 7.0 g agar) for 24 h before performing transformations. Chloroplast transformation was performed as previously described [113], using a PDS 1000/He biolistic particle delivery system (Bio-Rad, Hercules, CA, USA). Briefly, the abaxial surface of leaves were bombarded with 0.7 µm tungsten particles coated with 1 µg plasmid DNA using 1100 psi rupture discs at a 6 cm bombardment distance (Bio-Rad). Primary spectinomycin-resistant shoots were obtained after about six weeks on the RMOP regeneration medium containing 500 mg/l spectinomycin. To obtain homoplasmic plants, independent transplastomic lines were subjected to three subsequent regeneration rounds on antibiotic selection medium. Resistant shoots were also evaluated on a double selection medium containing 500 mg/l each of streptomycin and spectinomycin to prevent the selection of spontaneous spectinomycin mutants. After PCR and Southern blot confirmation, homoplasmic plants were rooted in the MS medium containing 500 mg/l of spectinomycin. Well-rooted plants were acclimated to greenhouse conditions.

### Molecular analysis

Total cellular DNA was extracted from transplastomic and wild-type *N. tabacum* plants according to the CTAB method [114] for PCR and Southern blot analysis. PCR amplification was performed to confirm the integration of the DARPin G3 gene into the *N. tabacum* chloroplast genome and the transplastomic nature of the regenerated shoots. A pair of DARPin G3-specific primers (forward primer (P1) 5′-GCTGCTAGAGCTGGACAAGA-3′ and reverse primer (P2) 5′-TGTCCAATAAAAGCAGCTAAATGT-3′) were used to confirm the integration of the DARPin G3 gene into the chloroplast genome. PCR analysis resulted in an amplificant of 215 bp as part of the DARPin G3 gene. One pair of primers (forward primer (P3): 5′- AACTAAACACGAGGGTTGC-3′ and reverse primer (P4): 5′-AGTATTAGTTAGTGATCCCGAC-3′) was used to detect a portion of the *rrn16*/*rps7/12* sequence along with the *aadA* and DARPin G3 genes. According to the mechanism of homologous recombination, it was predicted that only a ~ 2 kb PCR-amplified product could be detected in wild-type plants, while a ~ 4 kb fragment from transplastomic plants was anticipated due to the insertion of the expression cassette. Therefore, both fragments could be amplified in heteroplasmic plants, whereas only the ~ 4 kb fragment would be observed in homoplasmic plants. The positions of primers in the tobacco chloroplast genome are indicated in Fig. 1.

For Southern blot analysis, 2–3 g of transplastomic and wild-type tobacco plants DNA were digested with *Bgl*II, separated by gel electrophoresis in 0.8% agarose gels, and transferred by capillary blotting onto a positively charged nylon membrane (Roche applied science, Germany) using standard protocols [115]. A hybridization procedure according to the instruction manual of the DIG DNA labelling and detection kit (Roche applied science, Germany) was used, and 232 bp DNA fragments derived from the *rrn16* flanking sequence served as probes (Fig. 1). The probe was labelled with DIG-dUTP using the PCR DIG Probe Synthesis kit (Roche Applied Science, Germany). The homoplasmic tobacco plants were selected for further protein analysis.

### Isolation of chloroplasts

The chloroplasts of transplastomic and wild-type tobacco plant leaves were isolated for preparing chloroplast protein used in downstream analysis. The leaves of transplastomic and wild-type plants were finely grounded and homogenized in 3 volumes (v/w) of ice-cold chloroplast isolation buffer (50 mM Tris–HCl, 0.35 M mannitol, 5 mM disodium EDTA, 0.1% BSA (w/v), and 1.0 mM 2-mercaptoethanol) using a motor-driven blender. After passing the homogenate through three layers of Miracloth, it was centrifuged at 4 °C for 20 min at 200 × *g* to pellet cell debris and nuclei. The supernatant was decanted into new centrifuge tubes and centrifuged at 4 °C for 20 min at 1000 × *g* for the isolation of chloroplasts. The supernatant was rejected, and the green pellet was washed twice by resuspending in the isolation buffer and centrifuged at 1500 × *g* for 10 min. The supernatant was rejected, and the chloroplast pellet was used for protein extraction.

### Protein extraction and Western blot analyses

For the Western blot analysis, the total soluble protein was extracted from transplastomic tobacco leaves and isolated chloroplasts. The grounded leaves in the presence of liquid nitrogen or isolated chloroplasts were resuspended in 5 volumes of cold extraction buffer (PBS 1 × , pH 7.4, 150 mM NaCl, 1 ×  protease inhibitor), vigorously vortexed, and incubated for 30 min at 4 °C. The extracts were then centrifuged at 13,000 rpm for 10 min at 4 °C. Supernatants were collected, and total protein concentrations were determined using the Bradford protein assay [116].

Western blotting was carried out for the determination of (HE)_3_-DARPin G3 in transplastomic plants. Following the boiling of soluble protein samples with sample buffer at 95 °C for 5 min, the equal concentration of total soluble proteins from leaves and isolated chloroplasts were separated on a 12% sodium dodecyl sulfate–polyacrylamide gel in reducing conditions. After gel electrophoresis, the proteins were electro-transferred onto a PVDF (polyvinylidene difluoride) membrane (Bio-Rad) using Mini Transblot (Bio-Rad) following the manufacturer's instructions. The immunologic detection of (HE)_3_-DARPin G3 protein bound to PVDF membrane was performed using rabbit anti-His-tag antibody as primary antibody and goat anti-rabbit conjugated with horseradish peroxidase (HRP) antibody as secondary antibody and the addition of DAB-peroxidase substrate solution. The total soluble protein extracted from wild-type plants was used as the negative control.

### Quantitative enzyme-linked immunosorbent assay (ELISA) analysis

DARPin G3 expression levels in transplastomic plants were quantified by ELISA. ELISA 96-well microplates were incubated overnight at 4 °C with 50 ng/well of total soluble proteins of leaves and isolated chloroplasts of transplastomic and wild-type plants in three replicates. After three washes with PBS-T (PBS + 0.1% Tween 20), the nonspecific binding was blocked for 2 h at 37 °C with 300 µl per well of blocking buffer (1% BSA in 1X PBS buffer + 0.1% Tween 20). After washing as above, rabbit anti-His-tag antibody diluted to 1:1000 with blocking buffer was added to the wells and incubated for 2 h at 37 °C. After washing as above, the goat anti-rabbit conjugated with HRP in blocking buffer was added to each well at a dilution of 1:10,000. This was incubated for 2 h at 37 °C before being washed three times with PBS-T buffer. The color was developed using 100 μL of a 3,3′,5,5′-Tetramethylbenzidine (TMB) peroxidase substrate solution (200 mM citrate buffer, pH 3.95 + 1% TMB + 0.01% H_2_O_2_) at ambient temperature in the dark to maximize the reaction rate. The reaction was stopped with 2 M H_2_SO_4_, and then the optical density of each well was detected at a wavelength of 450 nm by an ELISA reader. For a standard curve, purified 15 kDa His-tagged standard protein was applied in serial dilution (3 to 25 ng/ml) to the microplate and processed as above. The amount of DARPin G3 protein in the total soluble proteins of tobacco chloroplasts was estimated from the standard curve. The DARPin G3 expression levels were calculated as a percentage of the total soluble protein.

### Purification of DARPin G3

The (HE)_3_-DARPin G3 was purified directly from the total soluble proteins extracted from transplastomic plant’s isolated chloroplast using the QIAexpress Ni–NTA Protein Purification System (QIAGEN). One advantage of DARPins is that they can be expressed at a high yield in a soluble form. Total soluble proteins were mixed with 4 × binding buffer (2 M NaCl and 2 ×  PBS, no imidazole) at a 4:1 ratio in batch mode and incubated at 4 °C for 2 h. Then the protein–resin complex was packed into a column for the washing and elution steps. The column was then washed with the wash buffer (0.5 M NaCl and 0.5X PBS, no imidazole) and eluted in 0.5 mL fractions with the elution buffer (0.5 M NaCl and 0.5 × PBS containing 200 mM imidazole). After the third elution, 10 mM DTT was added to the sample to prevent probable dimerization, and it was stored at − 20 °C. After that, the purified DARPin was tested by SDS–PAGE.

### Quantification of DARPin G3 binding affinity to HER2 by ELISA

A binding assay for quantifying the affinity of chloroplast-made DARPin G3 was performed in 96-well plates pre-coated with the extracellular domain of HER2 (HER2-ECD). The coating was performed with 100 μL of 1 μg/mL HER2-ECD (Sino Biological, 10,004-HCCH) and stored overnight at 4 °C. The plate was then washed twice with a phosphate-buffered saline solution containing 0.1% tween-20 (PBS-T) and blocked of non-specific binding sites on a shaker for 1 h at room temperature with PBS-T containing 1% BSA. The ELISA procedure employed a dilution series of purified chloroplast-made DARPin G3 ranging from 100 nM to 10 pM in PBS-T/BSA. Each dilution was administered in 100 μL volumes to duplicate wells and incubated at room temperature for 1 h with shaking. All wells were then washed three times with 200 μL of PBS-T. The extent of binding was determined by probing with a rabbit anti-His-tag antibody (1:1000 in PBS-T/BSA) which recognizes the N-terminal (HE)_3_-tag of the chloroplast-made DARPin G3, incubated for an hour on a shaker at room temperature. After three times washing with PBS-T, incubation with the goat anti-rabbit antibody conjugated with HRP in a final volume of 100 μL of PBS-T/BSA at a dilution of 1:10,000 for 1 h at room temperature on a shaker was done, and then each well was washed with 200 μL of PBS-T for triplet. The ELISA was developed with 100 μL of TMB liquid substrate per well and incubated at room temperature until a suitable colorimetric change occurred. The reaction was stopped by adding 100 μL of 2 N H_2_SO_4_ and absorbance readings were measured at 450 nm using an ELISA reader. To test whether the chloroplast-made DARPin G3 competed with trastuzumab for the epitope, 100 nM trastuzumab (Herceptin^®^, Roche) was used as a competitor for antigen binding in ELISA experiment. A crude extract of isolated chloroplasts of wild-type plants and BSA served as a negative control.

### Cancer cell culture

Three different breast cancer cell lines that have been described to express HER2 to varying extents were used to investigate chloroplast-made DARPin G3 specificity for HER2. SKBR-3, a HER2-positive breast cancer cell line that has been shown to express 10^6^ HER2 molecules per cell, MCF-7 cells, which only weakly express HER2, and the MDA-MB-231 breast cancer cell line, which hardly expresses HER2 at all, were obtained from the Immunology Research Center (Tabriz, Iran). All cell lines were cultured in RPMI 1640 medium containing 1% penicillin (10,000 units/ml), 1% streptomycin (10 mg/ml), and 10% fetal bovine serum (FBS) in a humidified incubator with 5% CO_2_.

### Flow cytometry

Flow cytometry was performed to investigate the binding specificity of the chloroplast-made DARPin G3 to HER2 on different breast carcinoma cell lines. All cell lines were individually prepared for flow cytometry according to the previously described method with a little modification [25]. Briefly, the medium was removed and the cells were incubated for 10 min with 5 ml of 0.2% EDTA. The cells were then transferred to tubes and centrifuged at 1000 rpm for 5 min at 4 °C. The EDTA-containing supernatant was removed, and 5 ml of fresh medium was added. For each test condition, 1 ml of cells that had been counted and diluted to 10^6^ cells per ml was employed. After washing with cold PBS containing 1% BSA, chloroplast-made DARPin G3 bearing N-terminal (HE)_3_-tag was added to cells to a final concentration of 100 nM and incubated for 1 h at 4 °C. Cells were washed with cold PBS/BSA and incubated with 200 μl of PBS/BSA containing rabbit anti-His-tag antibody at a dilution of 1:1000 for 1 h at 4 °C. Subsequently, the cells were washed with cold PBS/BSA and incubated with 200 μl of fluorescein isothiocyanate-coupled donkey anti-rabbit IgG at a dilution of 1:1000 for 30 min at 4 °C. The cells were washed with cold PBS and resuspended in 500 μl of cold PBS. Samples were analyzed on a MACSQuant 10 Flow Cytometer (Miltenyi Biotec, Germany); measurements were performed at a flow rate of 500 s^−1^. After being excited with an argon laser at 488 nm, fluorescence was detected at 525 nm. Cells were gated based on size scattering, forward scattering, and pulse width, so only single cells were analyzed. The cells without treatment or treated with anti-his tag antibody followed by FITC donkey anti-rabbit IgG were used as negative groups. A total of 10,000 cell events were recorded per sample. The software FlowJo (Tree Star, Ashland, OR) was used to analyse the data.

### Immunofluorescent microscopy

First, 1.0 × 10^4^ cells were seeded on sterile 96-well culture plates and incubated overnight at 37 °C and 5% CO_2_. The medium was removed and the cells were washed with ice-cold PBS containing 1% BSA. Cells were treated with 200 nM chloroplast-made DARPin G3 in PBS/BSA for 1 h at 4 °C. After three washes with cold PBS/BSA, the cells were incubated for 1 h at 4 °C with rabbit anti-His-tag antibody (1:1000 diluted in PBS/BSA) as the primary antibody. Cells were washed and incubated with FITC donkey anti-rabbit IgG (1:1000 diluted in PBS/BSA) as a secondary antibody for 30 min at 4 °C in the dark. After three times washing with cold PBS, cells were analyzed using the Citation 5 Cell Imaging Multimode Reader (BioTek, Winooski, VT). The laser at 469 nm excited the DARPin G3-FITC. The fluorescence of FITC was registered in the 525 nm.

## Data Availability

The nucleotide and protein sequence data of the expression cassette of chloroplastic DARPin G3 are available in GenBank at NCBI under the accession number ON186655. The datasets used and/or analyzed during the current study are available from the corresponding author on reasonable request.

## Abbreviations

BAP: 6-Benzylaminopurine
BSA: Bovine serum albumin
bp: DNA base pair
DNA: Deoxyribonucleic acid
ELISA: Enzyme-linked immunosorbent assay
FITC: Fluorescein isothiocyanate-coupled
HER2: Human epidermal growth factor receptor 2
HRP: Horseradish peroxidase
NAA: 1-Naphthaleneacetic Acid
PCR: Polymerase chain reaction
SDS–PAGE: Sodium dodecyl sulphate–polyacrylamide gel electrophoresis
TMB: 3,3′,5,5′-Tetramethylbenzidine
